# Retrospective observational study of the magnetic resonance imaging features of *MPV17*-related mitochondrial DNA depletion syndrome

**DOI:** 10.1007/s00247-025-06341-z

**Published:** 2025-08-07

**Authors:** Suzanne O’Hagan, Surita Meldau, Penelope Rose, Magriet Van Niekerk, Christelle Ackermann, Gillian Riordan, Ronald Van Toorn

**Affiliations:** 1 Division of Radiodiagnosis, Faculty of Medicine and Health Sciences, Stellenbosch University and Tygerberg Hospital, Francie Van Zijl Drive, Parow, Cape Town, 7505 South Africa; 2 National Health Laboratory Service, Johannesburg, South Africa; 3 University of Cape Town, Rondebosch, South Africa; 4 Red Cross War Memorial Children’s Hospital, Cape Town, South Africa

**Keywords:** Mitochondrial disease, Mitochondrial DNA, MPV17 mitochondrial DNA depletion syndrome, Neuroimaging, Pediatric, Resource-limited settings, Reticular formation

## Abstract

**Background:**

*MPV17*-related mitochondrial deoxyribonucleic acid (DNA) maintenance defects present in most affected individuals as an early-onset encephalohepatopathic disease. Diagnosis requires comprehensive molecular genetic testing, which is often not available in resource-limited settings. Therefore, the role of imaging as a diagnostic tool necessitates further exploration. Herein, we present the largest known cohort of patients with genetically confirmed *MPV17*-related mitochondrial DNA depletion syndrome, highlighting in detail the neuroimaging findings.

**Objective:**

To establish novel features on magnetic resonance imaging (MRI) that characterise *MPV17*-related mitochondrial DNA depletion syndrome, in order to provide a non-invasive, accessible, and reproducible biomarker inquiry.

**Materials and methods:**

Retrospective, descriptive study based at a large tertiary level hospital. Eight patients with *MPV17*-related mitochondrial DNA depletion syndrome who had undergone brain MRI were identified between 2015 and 2023. Neuroimaging findings were captured and described in detail. Two board-certified radiologists with experience in paediatric neuroradiology reviewed all images by consensus.

**Results:**

All patients were homozygous for the *MPV17*: c.106C>T variant. Age at brain MRI ranged from 11 days to 8 months. Seven out of the eight patients showed signal abnormalities in the reticulospinal tracts and/or reticular formation. Other neuroimaging findings included leukoencephalopathy, injury to extra-reticular white matter tracts and frequent basal ganglia involvement. Newly identified areas of involvement include the perirolandic cortices, hippocampi, optic pathways and olfactory nerves.

**Conclusion:**

Lesions in the reticular formation and reticulospinal tracts on brain MRI in a neonate or infant with hepatic dysfunction may represent a distinctive, albeit not specific, feature of *MPV17*-related mitochondrial DNA depletion syndrome.

**Graphical Abstract:**

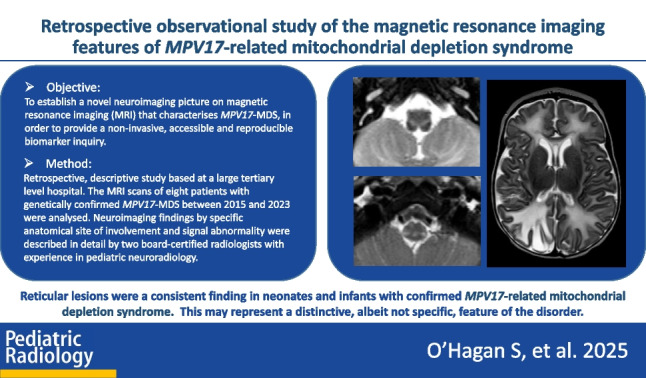

## Introduction

*MPV17*-related mitochondrial DNA depletion syndrome (MPV17-MDS) is a rare, congenital, autosomal recessive primary mitochondrial disorder characterised by early-onset hepatopathy and neurologic manifestations. It is caused by pathogenic variants in the *MPV17* gene, which normally encodes a non-selective channel located in the inner mitochondrial membrane [1–3]. Although the exact function of the *MPV17* channel remains unclear, its absence results in significant mitochondrial DNA (mtDNA) depletion, leading to an energy crisis in various cell types, most notably the liver.

*MPV17*-related mitochondrial DNA depletion syndrome accounts for the majority of nuclear DNA inherited mitochondrial disease known in Sub-Saharan Africa, due to the presence of a common pathogenic variant in *MPV17*, namely c.106C>T [4]. This variant has a predicted carrier frequency of one in 68 (95% CI 1 in 122 to 1 in 38) in the black South African population in the Western Cape and an estimated newborn incidence of one in 18,622 births (95%CI 1 in 59 to 1 in 5,776) in the same population [5].

Presentation most often occurs during the neonatal period (38%) or infancy (58%), with a typical limited life span of only a few months [2, 6]. Childhood onset has been reported in rare instances [7]. Liver dysfunction occurs early, with neurological manifestations generally being mild at the onset of the disease [8]. The clinical manifestations, in order of frequency, are liver dysfunction, liver failure, cholestasis, developmental delay, failure to thrive, hypotonia, microcephaly, lactic acidosis, hypoglycaemia and gastrointestinal dysmotility. Lesser common (<20%) manifestations include peripheral neuropathy, seizures, dystonia, ataxia, liver cirrhosis, renal tubulopathy, nephrocalcinosis, hypoparathyroidism, retinopathy and corneal ulcers. The prevalence of neuropathy may be underdiagnosed, as affected individuals often develop distal weakness only in later life. Bony changes associated with *MPV17* mutations include osteoporosis, osteopenia, scoliosis, platyspondyly and short stature [3]. Life expectancy in cases with infantile onset (0–1 year) is usually less than 5 years, depending on age of onset, severity of symptoms and rate of disease progression.

Neuroimaging plays an important role in the investigation and management of patients with mitochondrial disorders, in some cases providing non-invasive and reproducible biomarker inquiry. The existing MRI literature on *MPV17*-related mitochondrial DNA depletion syndrome is limited and lacks specificity. Radiological findings reported in case studies include white matter signal changes, abnormal myelination, and signal alterations in the brainstem and basal ganglia. Specific anatomical regions of the brain reportedly affected are non-unifying and include cerebellar white matter, middle cerebral peduncles, substantia nigra and dorsal brainstem [9]. Notably, no study has identified a consistent or specific pattern of involvement, except for a case report on siblings and three children from Saudi Arabia, which showed nearly identical signal changes in the reticulospinal tracts and reticular formation on MRI [10, 11].

This review aims to provide an overview of the genetic and clinical features of *MPV17*-related mitochondrial DNA depletion syndrome, accompanied by a detailed pictorial description of neuroimaging findings in eight affected infants, and a discussion of the key findings and their diagnostic implications.

## Methods

This retrospective descriptive study was based at a large tertiary level hospital. Eight patients (five male) with genetically confirmed *MPV17*-related mitochondrial DNA depletion syndrome (encephalohepatopathy form) who underwent brain MRI were identified between 2015 and 2023. MRI was performed on a 1.5-T MAGNETOM Aera scanner (Siemens Healthineers, Erlangen, Germany). Six patients required general anaesthesia, and the other two were already intubated and ventilated. The standard MRI protocol included axial T1WI, axial and coronal T2WI, axial fluid-attenuated inversion recovery (FLAIR), diffusion-weighted imaging (DWI) and apparent diffusion coefficient (ADC) mapping, susceptibility-weighted imaging (SWI), and magnetisation-prepared rapid acquisition with gradient echo (MP-RAGE). Contrast was administered in five patients, although the specific indications for this are unknown. Magnetic resonance spectroscopy (MRS) was included in one protocol at the request of a clinician, using a short echo time (TE) of 30 ms with single-voxel acquisition. The neuroimaging findings were captured and described by specific anatomical site of involvement and signal abnormality. Two board-certified diagnostic radiologists with 8 years and 18 years of experience in adult and paediatric neuroradiology, respectively, reviewed all images by consensus. In addition to radiological data, demographic data, including patient age at diagnosis and MRI, sex, and clinical manifestations, were collected.

## Results

### Clinical presentation

All patients were homozygous for the *MPV17*: c.106C>T variant. The mean patient age at diagnosis was 10 weeks, with a range of 1 week to 32 weeks. The ages of the patients at the time of brain MRI ranged from 11 days to 32 weeks. There were no cases of familial consanguinity. All symptomatic infants presented with severe failure to thrive, characterised by weight, length and weight-for-height below the third percentile. Additional symptoms included cholestatic jaundice and gross motor delay. Physical examination findings consisted of central hypotonia (poor head control) with weakness (resulting in frog-like posture), reduced peripheral deep tendon reflexes and hepatomegaly, with or without splenomegaly. Microcephaly (head circumference below the third percentile for age and sex) was observed in two patients. Laboratory investigations revealed elevated transaminases and alkaline phosphatase, conjugated hyperbilirubinaemia, serum lactatemia and coagulopathy. Cerebrospinal fluid (CSF) lactate levels were assessed in all patients and found to be markedly elevated, exceeding 10 mmol/L. This is significantly higher than the normal reference range of 1.2–2.8 mmol/L. Cardiomyopathy, endocrinopathy, ophthalmological or bony abnormalities were not observed in any of the patients.

Neonatal manifestations included neonatal encephalopathy (*n*=3), characterised by lethargy and poor sucking, as well as neonatal seizures (*n*=2), jaundice and episodes of hypoglycaemia (*n*=3). One case presented with subtle seizures (lip-smacking), while the other exhibited tonic seizures. All patients were full-term babies with normal birth weights and unremarkable perinatal histories, except one patient who was preterm (32 weeks), human immunodeficiency virus (HIV) exposed but uninfected, with low birthweight for gestational age. The mother was on antiretroviral therapy, and the hyperlactatemia was initially attributed to this treatment. There was no relevant family history (early, unexplained deaths or miscarriages) in any of the families.

Out of the eight patients, five demised, two were lost to follow-up (status is unknown), and one child is still alive (age 62 weeks). The mean survival time was 39.6 weeks (range 5–70 weeks).

## Magnetic resonance imaging brain findings by age, anatomical location, and sequence-specific signal abnormality (Table 1)

**Table 1:** Magnetic resonance imaging brain findings by anatomical location

Case	Age at MRI	Reticular formation and reticulospinal tracts	Basal ganglia and thalami	Brainstem^a^	Supratentorial White matter	Hippocampi	Perirolandic cortex	Restricted diffusion	Other
1	**11 days**	Yes	No	Yes Rubrospinal tracts midbrain	Yes Central corticospinal tracts	No	Yes T1 ↑ DWI +	Yes	No other evidence of HII
2	**2 weeks**	Yes T2 ↑ DWI-	Yes Caudate heads Globus pallidi	Yes Cerebral peduncles, midline tegmentum, medullary pyramidal tracts	Yes Corticospinal tracts	No	Yes T1 ↑ DWI +	Yes	No other evidence of HII Microcephaly
3	**5 weeks**	Yes T2 ↑ DWI-	No	No	Yes Diffuse WM, asymmetric, dorsal gradient, PLIC	No	No	No	Preterm 32 weeks Focus of blooming on SWI No features of PVL
4	**3 months**	No	No	No	No	No	No	No	Normal MRI No clinical encephalopathy
5	**4 months**	Yes T2 ↑ DWI-	Yes Globus pallidi—posteromedial	No	No	No	No	No	N/A
6	**5 months**	Yes T2 ↑ DWI-	Yes Globus pallidi Pulvinars	No	No	No	No	Yes	Microcephaly Restricted diffusion fornices, optic chiasm, tracts and olfactory nerves
7	**8 months**	Yes T2 ↑ DWI-	Yes Globus pallidi—medial	Yes Cerebral peduncles	Yes Diffuse WM, dorsal gradient, PLIC, splenium	Yes	No	Yes	Restricted diffusion fornices, optic chiasm, tracts and olfactory nerves
8	**8 months**	Yes T2 ↑ DWI + (central tegmental tracts)	Yes Globus pallidi—posteromedial	Yes Cerebral peduncles	Yes Diffuse WM, symmetric, dorsal gradient PLIC, external capsule, fornices	Yes	No	Yes	Restricted diffusion fornices, optic chiasm, tracts, olfactory nerves and cingulate gyri

*DWI* diffusion-weighted imaging, *HII* hypoxic ischemic injury, *HIV* Human Immunodeficiency Virus, *MRI* magnetic resonance imaging, *PLIC* posterior limb of internal capsule, *PVL* periventricular leukomalacia, *SWI* susceptibility-weighted imaging, *WM* white matter

^a^Excludes sites within the reticular formation

### Reticular formation and reticulospinal tracts

**Fig. 1:**
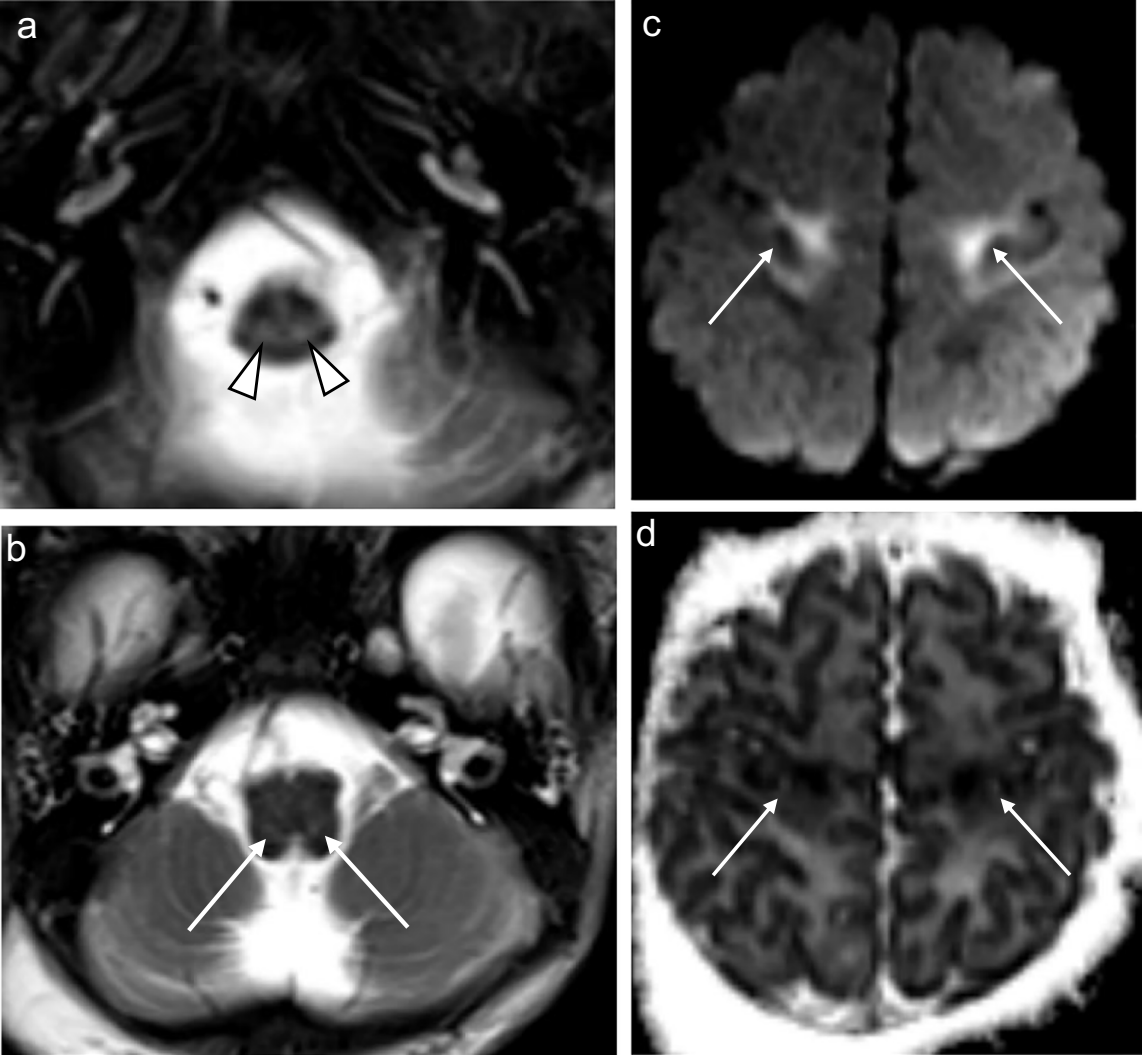
An 11-day-old boy with genetically confirmed *MPV17*-related mitochondrial depletion syndrome. **a** Axial T2-weighted image shows hyperintense signal in the reticulospinal tracts of the proximal cervical cord (*arrowheads*). **b** Axial T2-weighted image shows hyperintense signal in the reticular formation in the medulla (*arrows*). **c** Axial diffusion-weighted image and (**d**) apparent diffusion coefficient map demonstrate restricted diffusion in the perirolandic cortex (*arrows*)

**Fig. 2:**
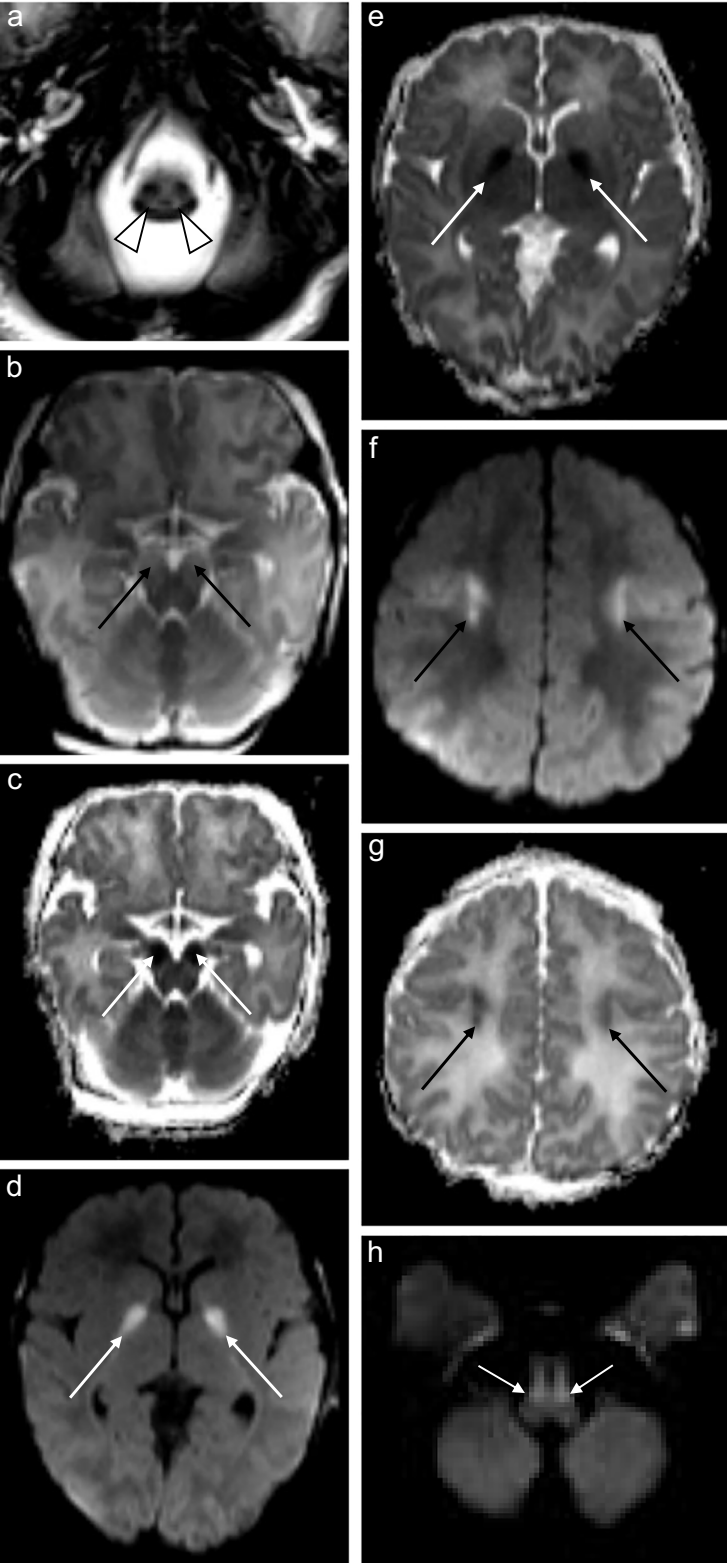
A 2-week-old boy with genetically confirmed *MPV17*-related mitochondrial depletion syndrome. **a** Axial T2-weighted image shows hyperintense signal in the reticulospinal tracts of the proximal cervical cord (*arrowheads*). **b** Axial diffusion-weighted image and (**c**) apparent diffusion coefficient map demonstrate restricted diffusion in the cerebral peduncles (*arrows*). **d** Axial diffusion-weighted image and (**e**) apparent diffusion coefficient map demonstrate restricted diffusion in the globus pallidi (*arrows*). **f** Axial diffusion-weighted image and (**g**) apparent diffusion coefficient map demonstrate restricted diffusion in the subperirolandic white matter (*arrows*). **h** Axial diffusion-weighted image shows restricted diffusion in the medullary pyramidal tracts (*arrows*)

**Fig. 3:**
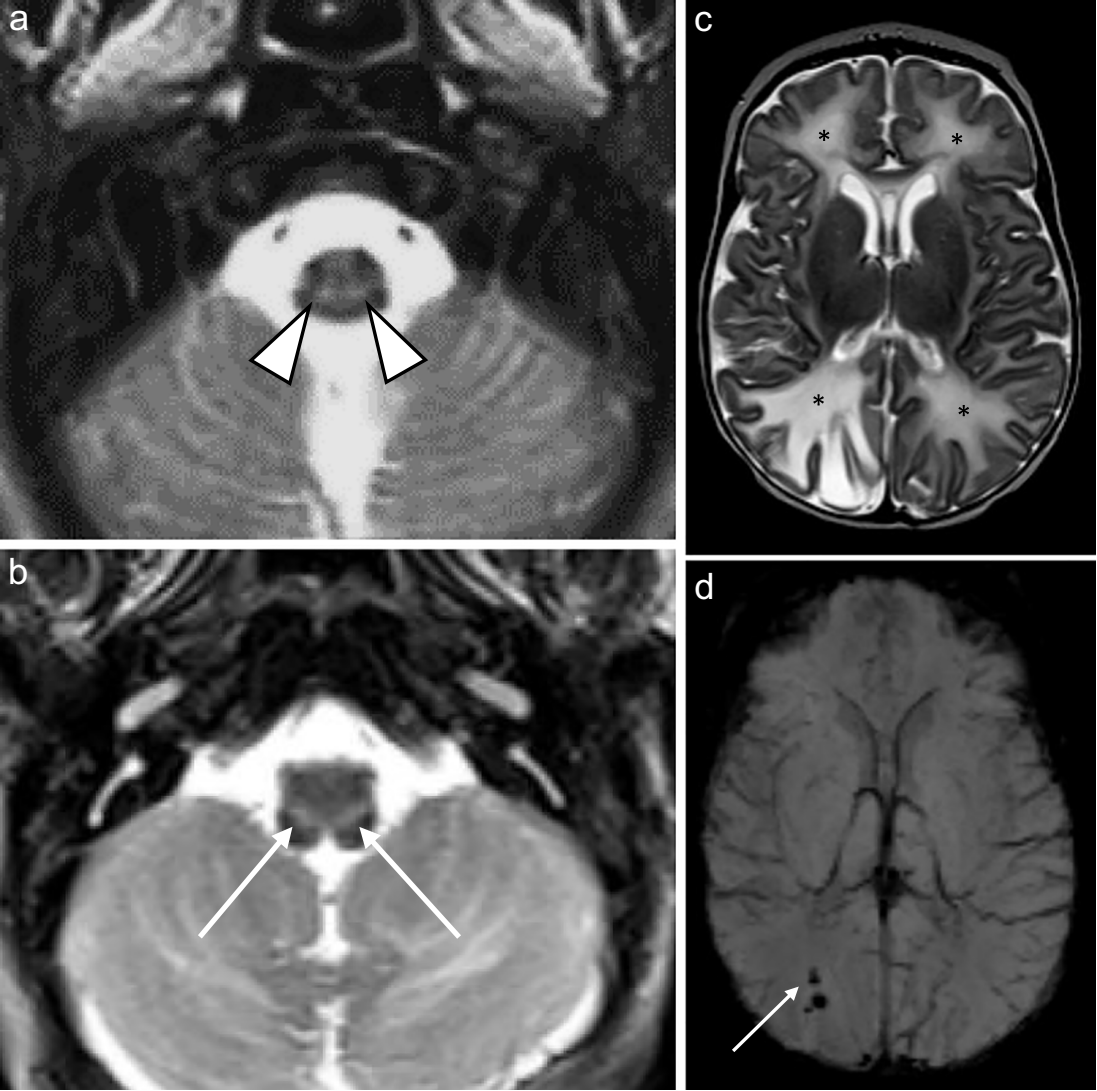
A 5-week-old girl with genetically confirmed *MPV17*-related mitochondrial depletion syndrome. **a** Axial T2-weighted image shows hyperintense signal in the reticulospinal tracts of the proximal cervical cord (*arrowheads*). **b** Axial T2-weighted image shows hyperintense signal in the reticular formation of the medulla (*arrows*). **c** Axial T2-weighted image shows extensive white matter disease involving the periventricular, deep, and subcortical white matter with extension into the U-fibers (*asterisks*). **d** Susceptibility-weighted imaging reveals blooming at the site of maximally diseased white matter (*arrow*)

**Fig. 4:**
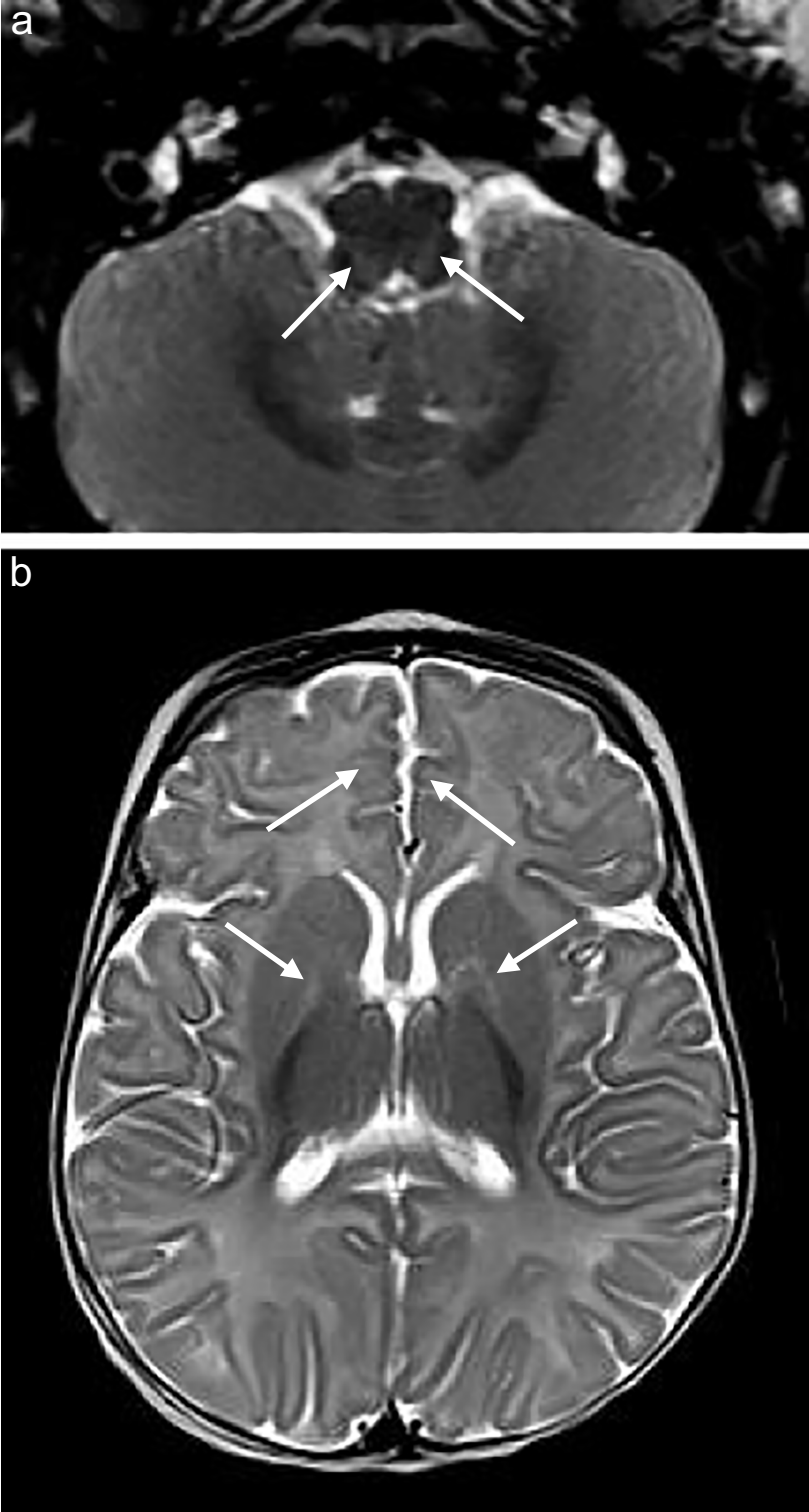
A 4-month-old female with genetically confirmed *MPV17*-related mitochondrial depletion syndrome. **a** Axial T2-weighted image shows hyperintense signal in the reticular formation of the medulla (*arrows*). **b** Axial T2-weighted image shows hyperintense signal in the posteromedial globus pallidi (*arrows*)

**Fig. 5:**
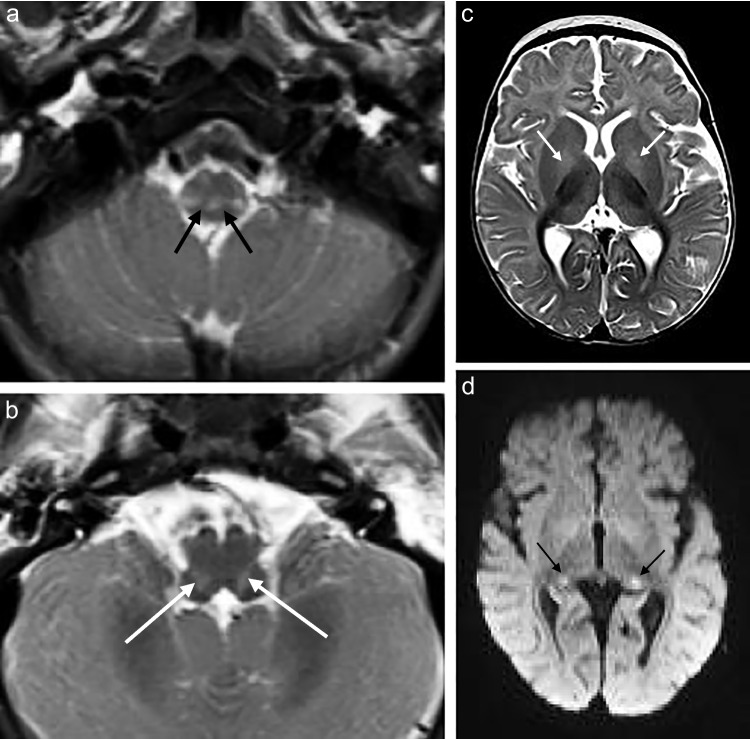
A 5-month-old male infant with genetically confirmed *MPV17*-MDS. **a** Axial T2-weighted image shows hyperintense signal in the reticular formation of the proximal cervical cord (*arrows*). **b** Axial T2-weighted image shows hyperintense signal in the reticular formation of the medulla (*arrows*)*.* **c** Axial T2-weighted image shows hyperintense signal in the globus pallidi. **d** Axial diffusion -weighted imaging shows restricted diffusion in the thalamic pulvinars (*arrows*)

**Fig. 6:**
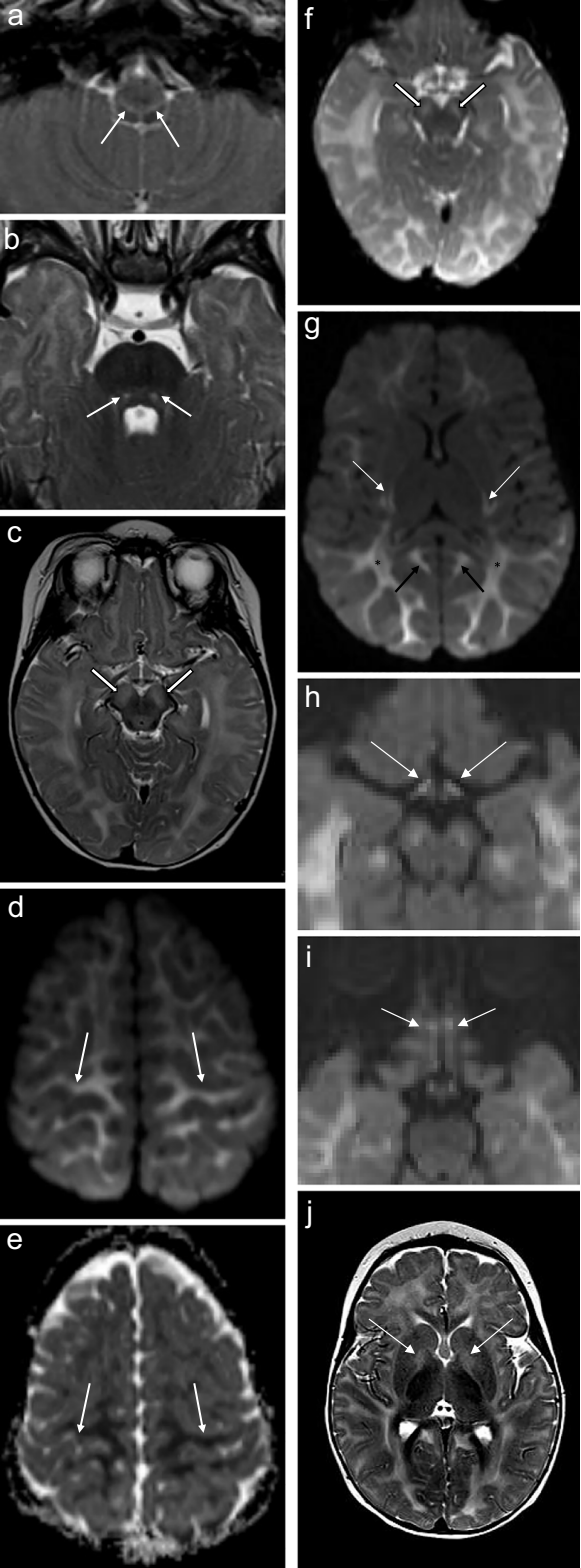
An 8-month-old girl with genetically confirmed *MPV17*-related mitochondrial depletion syndrome. **a** Axial T2-weighted image shows hyperintense signal in the reticular formation of the medulla (*arrows*). **b** Axial T2-weighted image shows hyperintense signal in the central tegmental tracts of the pons (*arrows*). **c** Axial T2-weighted image shows hyperintense signal in the cerebral peduncles of the midbrain (*arrows*). **d** Axial diffusion-weighted imaging and (**e**) apparent diffusion coefficient maps confirm restricted diffusion symmetrically present in the subperirolandic white matter (*arrows*). **f** Axial diffusion-weighted imaging shows restricted diffusion in the cerebral peduncles (*arrows*). **g** Axial diffusion-weighted imaging shows restricted diffusion present within the white matter (*asterisks*), posterior limbs of the internal capsules (*white arrows*) and cingulate gyri (*black arrows*). **h*** DWI *image shows restricted diffusion in the optic tracts and olfactory nerves (*arrows*) (i). **j** Axial T2-weighted image shows hyperintense signal in the medial globus pallidi (*arrows*)

Seven of the eight patients who underwent MRI of the brain (88%) showed increased T2 and FLAIR signal conforming to the reticulospinal tracts and/or reticular formation, with one of these cases also exhibiting restricted diffusion on DWI/ADC (Figs. 1, 2, 3, 4, 5 and 6). The one case that did not show this finding was imaged at 3 months of age. This patient presented at 4 weeks of age with cholestatic jaundice, central hypotonia, bicytopenia, and lactataemia. Despite the absence of abnormalities on MRI, it is possible that the characteristic changes may have been delayed due to the absence of clinically detected encephalopathy. In contrast, all the other *MPV17* mitochondrial DNA depletion syndrome cases with encephalopathy exhibited abnormalities in the reticulospinal tracts and/or reticular formations. Notably, a neonate imaged on day 11 of life already showed reticulospinal tract pathology, suggesting a potentially early, or even antenatal onset of pathology.

### Supratentorial white matter

**Fig. 7:**
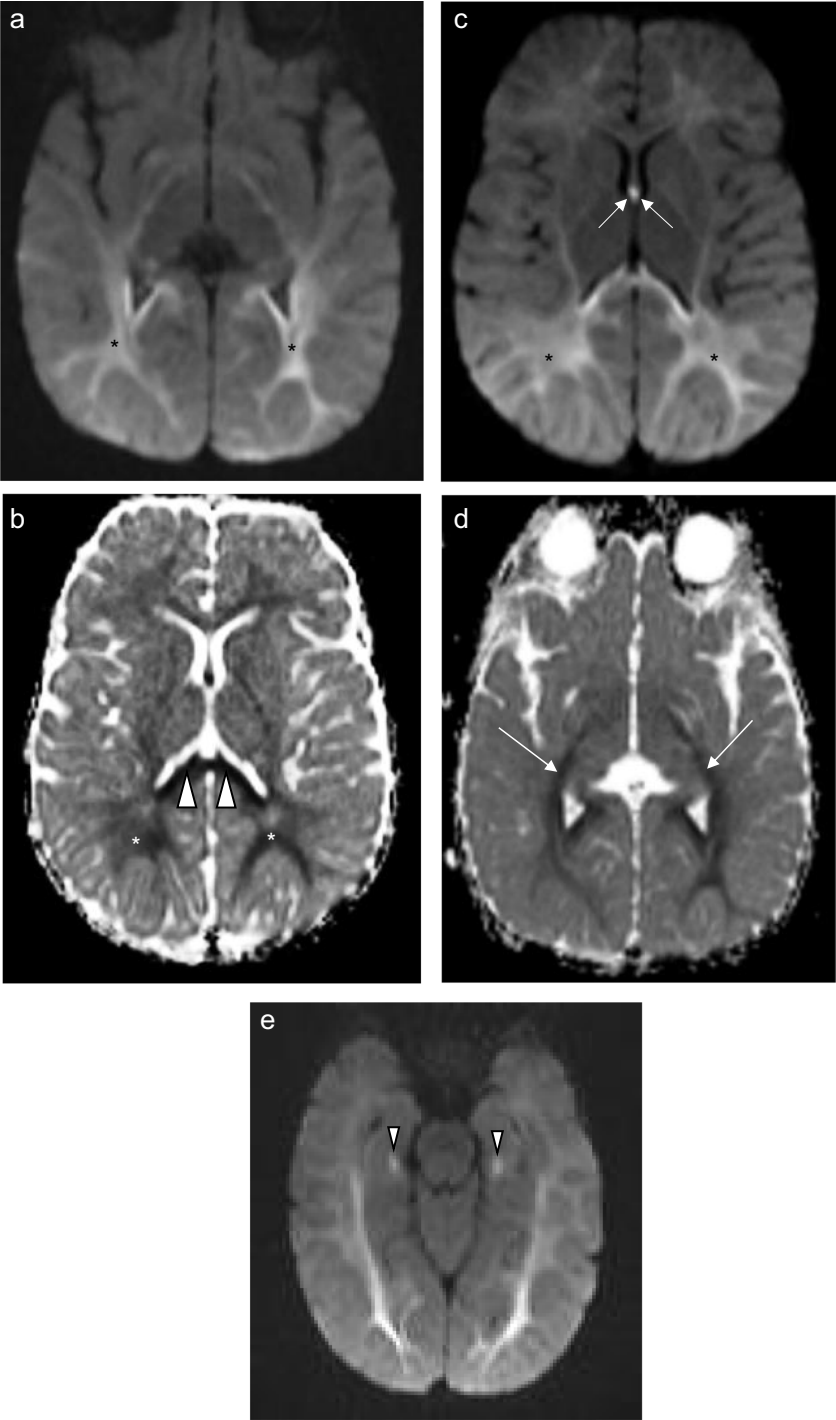
An 8-month-old boy with genetically confirmed *MPV17*-related mitochondrial depletion syndrome. (**a**) Axial diffusion-weighted imaging and (**b**) apparent diffusion coefficient map show posterior-predominant restricted diffusion symmetrically present in the white matter (*asterisks*) with associated involvement of the splenium of the corpus callosum (*arrowheads*). (**c**) Axial diffusion-weighted imaging further highlighting the posterior gradient of white matter disease (*asterisks*) and involvement of the fornices (*arrows*). (**d**) Apparent diffusion coefficient map involvement of the corticospinal tracts (*arrows*). (**e**) Axial diffusion-weighted imaging shows restricted diffusion in bilateral hippocampi (*arrowheads*)

Supratentorial white matter disease was detected in five of eight (62.5%) patients (Figs. 2, 3, 6 and 7). Both neonates demonstrated T2 hyperintensity and restricted diffusion in the central corticospinal tracts (sub-perirolandic, corona radiata), extending more caudally into the posterior limbs of the internal capsule in one case (Figs. 1 and 2). The 5-week-old preterm infant with a corrected gestational age of 37 weeks presented radiologically as diffuse, asymmetrical leukoencephalopathy with a dorsal gradient, characterised by T2 hyperintensity in the periventricular, deep, and subcortical white matter which extended into the U-fibres (Fig. 3). No normal T1 hyperintense myelin was present in the immature brain. A focus of blooming appeared on susceptibility-weighted imaging in the maximally affected right occipital lobe white matter. The MRI study of the second 8-month-old revealed diffuse leukoencephalopathy, again with a dorsal gradient, characterised by diffusion restriction in the subcortical white matter, posterior limbs of the internal capsules, and dorsal aspects of the external capsules and fornices (Fig. 6). The second 8-month-old infant manifested marked, posterior-predominant white matter diffusion restriction involving the posterior limbs of the internal capsules, and occipital more than frontal deep and subcortical white matter, as well as the splenium of the corpus callosum (Fig. 7). The neonates and 5-week-old had corpus callosum biometry within normal limits for age [12]. Evaluation of the callosal myelination status was confounded by the presence of leukoencephalopathy in most cases but there was no significant deafferentation atrophy at the time of imaging or developmental anomalies.

### Basal ganglia and thalamus

Signal and volumetric alterations in the basal ganglia were identified in five of eight (62.5%) patients (Figs. 2, 4, 5 and 6). One of the neonates had isolated, symmetrical lentiform nucleus involvement. The caudate heads were symmetrically oedematous with facilitated diffusion, while the globus pallidi, also swollen, displayed restricted diffusion. This may reflect different stages of evolution of the cerebral lesions with pseudonormalisation of DWI in the globus pallidi. The 5-month-old infant had symmetrical diffusion restriction in the thalamic pulvinars without accompanying basal ganglia changes. The 4-month-old, 5-month-old, and two 8-month-old children had MRI studies which demonstrated T2 hyperintense signal in the globus pallidi, confined to the medial or posteromedial aspects in three of the four cases.

### Brainstem

The brainstem was affected in four of eight (50%) patients and all signal changes were topographically localised to white matter tracts (Figs. 2 and 6). The 11-day-old had signal alteration conforming to the rubrospinal tracts in the midbrain. The 2-week-old had abnormal signal conforming to the corticospinal tracts traversing the cerebral peduncles of the midbrain and medullary pyramidal tracts of the ventral medulla. Both 8-month-old infants manifested T2 hyperintense signal in the cerebral peduncles as part of corticospinal tract involvement. Restricted diffusion accompanied white matter tract signal abnormality in all but one case.

### Perirolandic cortex

Signal alterations in the sensorimotor cortex were identified in the two neonates (Figs. 1 and 2). This was differentiated from hypoxic ischaemic injury based on clinical histories which excluded perinatal asphyxia, the absence of characteristic changes in the basal ganglia-thalamus complex (posterolateral putamina and ventrolateral thalami), and the presence of restricted diffusion which would confine any potential hypoxic ischaemic injury to the acute phase. Perirolandic signal abnormality did not extend into the paracentral lobule [13].

### Hippocampi

Hippocampal involvement was evident in the two 8-month-old children, characterized only by restricted diffusion in both cases (Fig. 7). In the one infant, the parahippocampal gyri were also affected.

### Restricted diffusion

Only one of the seven patients with T2 hyperintense changes in the reticular formation had associated restricted diffusion which localised to the central tegmental tracts of the pons. Restricted diffusion in the diseased parts of the brain accompanied most *extra-reticular* MRI abnormalities, occurring in five of eight patients (62.5%) (Figs. 1, 2, 5, 6 and 7). The ADC values ranged from 431 × 10^–6^ mm^2^/s to 845 × 10^–6^ mm^2^/s with a mean value of 579 × 10^–6^ mm^2^/s. The ADC values tended to be lowest when acquired in the diseased white matter.

### Cranial nerves and nuclei

Half of the patients (50%) showed signal disturbances in these regions. One neonatal MRI revealed restricted diffusion in the midline tegmentum, near the oculomotor nuclei of the midbrain (Fig. 2). Three of the older infants’ (5 months to 8 months) MRI scans all demonstrated restricted diffusion in the optic chiasms and tracts, fornices, and olfactory nerves (Fig. 6).

### Other

Microcephaly was suspected in two patients (25%) based on sulcal prominence and reduction in craniofacial ratio, corresponding to the clinical finding of head circumferences below the 3rd centile. Apart from the preterm patient with diffuse T2 hyperintensity of the unmyelinated cerebellar white matter, no cerebellar abnormalities were detected. Contrast enhancement was not a feature of the disease when utilised. Magnetic resonance spectroscopy using a short echo time (TE) of 30 ms with single voxel acquisition was performed on one child and failed to demonstrate a glutamate or lactate peak, with results within normal range.

## Discussion

The clinical manifestations evident in our cohort of eight children are like those described in the literature, with severe failure to thrive, cholestatic jaundice, and gross motor delay being the most frequent symptoms.

All patients in our cohort were homozygous for a severe *MPV17* pathogenic variant, expected to result in no functional protein. It is therefore possible that cases with milder presentations caused by variants that only reduce or alter the *MPV17* channel’s functionality, rather than result in absence of the channel, may present differently. This could explain the discrepancies in severity of the various clinical manifestations and the inconsistent imaging presentations documented in the existing literature [9]. No information was available on the pathogenic variant affecting the siblings with reticular abnormalities described previously [10].

To our knowledge, the only cases published in the *radiological* literature are those of a sibling pair and three children from Saudi Arabia with variant-undisclosed *MPV17* who showed identical abnormalities of the reticulospinal tracts and reticular formation on 1.5-T MRI [10]. Our findings strongly corroborated the hypothesis that this anatomical subsite is consistently affected in *MPV17*-related mitochondrial DNA depletion syndrome patients, with seven out of eight of the patients in our series (and all encephalopathic patients) showing the same MRI abnormality.

The reticular formation is made up of a net-like structure of various brainstem nuclei and neurons and covers an expansive portion of the brainstem, beginning in the mesencephalon, extending caudally through the medulla oblongata, and projecting into the superior cervical spinal cord segments. The reticular formation does not have any distinct cytoarchitectural boundaries and is dispersed throughout the brainstem as a network of interconnected neurons with many projections cranially to subcortical and cortical brain structures as well as caudally to the spinal cord [14]. The reticulospinal tract is a descending tract present in the white matter of the spinal cord, originating in the reticular formation. It consists of bundles of axons that carry information from the reticular formation in the brainstem to the peripheral body parts, being primarily responsible for locomotion and postural control [15]. Since these structures reside at the craniocervical junction, which is located at the inferior edge of the field of view on standard brain MRIs, they may potentially be overlooked. For this reason, knowledge of this anatomical area and recognition of its relevance in *MPV17*-related mitochondrial DNA depletion syndrome are essential in directing the imaging search. With higher magnetic field imaging at 3-T becoming increasingly available, it is expected that signal changes to the reticular formation should be more readily observed.

In addition to our study reinforcing the significance of the reticular formation and reticulospinal tracts in *MPV17*-related mitochondrial DNA depletion syndrome, it further characterises the topography, frequency, and extent of extra-reticular findings, including a predilection for the basal ganglia, a tendency to produce severe leukoencephalopathy, and frequent involvement of the corticospinal tracts. We establish that restricted diffusion occurs almost always at extra-reticular sites of involvement but infrequently in the reticular formation. Novel areas of interest, not previously described in the literature, are the perirolandic cortices and hippocampal formations, both regions of high mitochondrial activity, as well as the optic tracts and olfactory nerves.

These findings contribute to the scarce body of literature pertaining to the neuroimaging manifestations of *MPV17*-related mitochondrial DNA depletion syndrome, despite the illness being largely defined by its neurological presentation. Since no specific patterns have been identified which would enable imaging to be used as a reliable diagnostic tool, these results establish a robust foundation to promote further exploration in this field [6, 7, 10, 16].

In cases where the perirolandic cortices in neonates and hippocampi in older infants were affected, there was no history of asphyxia, signal changes were acute, as evidenced by restricted diffusion, and there were no accompanying basal ganglia or thalamic changes to suggest hypoxic ischemic injury.

For those patients with genetically confirmed *MPV17*-related mitochondrial DNA depletion syndrome in whom initial neuroimaging is normal, follow-up MRI would enhance our understanding of the natural history of the condition. However, it may not always be feasible due to the rapidly progressive trajectory of this form of the disease, subjecting patients to early demise (mean age of death approximately 10 months) or the development of complications that preclude general anaesthetic. None of the patients in our cohort underwent follow-up neuroimaging.

An additional consideration is that most patients with *MPV17*-related mitochondrial DNA depletion syndrome present with cholestatic jaundice. It follows that hyperintense signal in the globus pallidi, identified in five of eight (62.5%) of the MRI studies, may indicate concurrent bilirubin encephalopathy, particularly in the two patients where the abnormality was linear and confined to the posteromedial aspects. The full diagnostic imaging triad of injury to the CA1 and CA2 sectors of the hippocampi and subthalamic nuclei was not, however, observed [17].

Furthermore, since the administration of contrast did not result in pathologic enhancement in any cases, it is probably not indicated when strong clinico-radiological evidence exists for *MPV17*-related mitochondrial DNA depletion syndrome.

This study has two primary limitations. Firstly, we did not compare our findings to other mitochondrial disorders or different variants of *MPV17*-related mitochondrial DNA depletion syndrome, which prevents us from determining the specificity of our results to *MPV17*-related mitochondrial DNA depletion syndrome. Secondly, despite being the largest single-centre, single-variant cohort in the literature, our patient population is still relatively small, limiting the statistical significance of our findings. In contrast, the literature on better-known mitochondrial disorders like Leigh syndrome has identified characteristic imaging manifestations. Spongiform lesions of the basal ganglia (particularly the putamina) and brainstem, with a predilection for the midbrain and periaqueductal grey matter, are commonly observed. A study published in AJNR in 2000 analysed the MRI findings of eight patients with known Leigh syndrome and found that half had lesions conforming to the lower brainstem, reticular formation, or reticulospinal tracts. The authors proposed that the detection of lower brainstem lesions at the onset of respiratory abnormalities might be specific for Leigh syndrome [18]. We now know that this is not the case and that such changes may be a manifestation of a broader range of mitochondrial conditions.

## Conclusion

This review underscores the consistent presence of reticular lesions in neonates and infants with confirmed *MPV17*-related mitochondrial DNA depletion syndrome. The signal abnormality within the reticular formation and/or reticulospinal tracts may be a distinctive, albeit not specific, feature of the disorder. This highlights the importance of including the often-overlooked reticular formation in imaging evaluations of neonates or infants presenting with hepatic and neurological dysfunction.

Notably, reticular lesions may be apparent within days of birth in severe cases presenting with neonatal encephalopathy, hypotonia, and seizures. Familiarity with these imaging biomarkers can facilitate expedited diagnosis, particularly in resource-limited settings where genetic testing is unavailable or delayed. These findings warrant further exploration of imaging changes in other mitochondrial disorders, potentially leading to improved diagnostic accuracy and patient outcomes.

While this study primarily highlights the selective vulnerability of the reticular formation and reticulospinal tracts to *MPV17*-related mitochondrial DNA depletion syndrome, it also reveals additional important, less specific findings. These include a predilection for the basal ganglia, corticospinal, and other extra-reticular white matter tracts, a tendency to produce severe leukoencephalopathy, frequent restricted diffusion in the affected brain regions, and involvement of previously undescribed areas, including the perirolandic regions, hippocampal formations, optic tracts, and olfactory nerves. Notably, MRI contrast agents may not be necessary when strong clinico-radiological evidence supports *MPV17*-related mitochondrial DNA depletion syndrome.

## Data Availability

No datasets were generated or analysed during the current study.
